# Efficient and Fast Differentiation of Human Neural Stem Cells from Human Embryonic Stem Cells for Cell Therapy

**DOI:** 10.1155/2017/9405204

**Published:** 2017-09-18

**Authors:** Xinxin Han, Liming Yu, Jie Ren, Min Wang, Zhongliang Liu, Xinyu Hu, Daiyu Hu, Yihong Chen, Li Chen, Ying Zhang, Yuehua Liu, Xiaoqing Zhang, Hua He, Zhengliang Gao

**Affiliations:** ^1^Shanghai Stomatological Hospital, Fudan University, Shanghai 200001, China; ^2^Shanghai Tenth People's Hospital, Tongji University School of Medicine, Shanghai 200092, China; ^3^School of Medicine, Jiaxing University, Zhejiang 314001, China; ^4^Changzheng Hospital, Second Affiliated Hospital of Second Military Medical University, Shanghai 200003, China; ^5^Shanghai Tenth People's Hospital, Tongji University School of Medicine, Tongji University Advanced Institute of Translational Medicine, Shanghai 200092, China

## Abstract

Stem cell-based therapies have been used for repairing damaged brain tissue and helping functional recovery after brain injury. Aberrance neurogenesis is related with brain injury, and multipotential neural stem cells from human embryonic stem (hES) cells provide a great promise for cell replacement therapies. Optimized protocols for neural differentiation are necessary to produce functional human neural stem cells (hNSCs) for cell therapy. However, the qualified procedure is scarce and detailed features of hNSCs originated from hES cells are still unclear. In this study, we developed a method to obtain hNSCs from hES cells, by which we could harvest abundant hNSCs in a relatively short time. Then, we examined the expression of pluripotent and multipotent marker genes through immunostaining and confirmed differentiation potential of the differentiated hNSCs. Furthermore, we analyzed the mitotic activity of these hNSCs. In this report, we provided comprehensive features of hNSCs and delivered the knowledge about how to obtain more high-quality hNSCs from hES cells which may help to accelerate the NSC-based therapies in brain injury treatment.

## 1. Introduction

Neurogenesis is defined as progress of new neuron generation from neural stem cells (NSCs) or usually named neural progenitor cells (NPCs) [1, 2]. Neurogenesis exists in both embryonic stages and adult stages. In adult, there are two distinct regions occurring neurogenesis in the central neural system (CNS): subventricular zone (SVZ) of lateral ventricles and the subgranular zone (SGZ) of the dentate gyrus in mammalian hippocampus [3, 4]. Embryonic neurogenesis taking place in the ventricular zone (VZ) and SVZ originates from the differentiation of neuroepithelial cells into radial glial cells (RGCs) in the mouse brain [4, 5].

Adult neurogenesis was firstly reported 50 years ago in the hippocampus of dentate brain (dentate gyrus, DG area) [6]. Before that, scientific community generally had believed for a long time that the adult brain cannot produce new neurons. Now, the idea is widely acknowledged that adult neurogenesis exists in the DG of human brain [7, 8]. Adult neurogenesis occurs in most mammals and several other vertebrates [9].

NSCs are multipotential stem cells with the capability to self-renew and can generate neurons, astrocytes, and oligodendrocytes [9]. NSCs play an important role both in basic research of neural development and wide potential for stem cell-based therapy in neurological diseases such as stroke, Parkinson's disease, and spinal cord injury [10, 11]. It has been reported that an immortalized human NSC line, HB1.F3 (F3), was constructed from a 15-week gestational human fetal brain but this cell line is overexpresses v-myc oncogene with a retrovirus vector [10]. Previous studies show that human NSCs which transplant by intravenous injection can differentiate into diverse neural cell types and reduce the neurological damage after stroke [12, 13].

At present, research on hES cells to neural differentiation is mainly focused on direct differentiation of mature functional neurons from hES cells or neural crest stem cells for clinical application [14, 15]. Noticeably, it is reported that a good manufacturing practice (GMP) differentiation procedure is devised for efficient production of dopamine progenitors from hES cells [16]. There is also research about obtaining GABA neurons from human embryonic stem cell [17] and cerebral cortex neurons by directing differentiation of human pluripotent stem cells [18]. Meanwhile, several groups successfully investigated that they can induce mature cortical neuron production from hES cells by applying some small molecular compounds [19–22].

Due to the potential of neural stem cells for cell therapy, the importance of developing and optimizing approaches was realized for production of hNSCs. Although the above studies can model cortical development well, most of the cells differentiated from hES cells are a mature mixed population including the upper layer and deep layer cortical neurons. It is unclear whether highly enriched hNSCs have been generated from hES cells. We like to develop differentiation protocols which eliminate the use of undefined factors.

Noggin, known as bone morphogenesis protein (BMP) inhibitor, is a critical neural-inducing factor both in frog and mammalian [23, 24]. Recombinant Noggin has been applied in different neural induction protocols for hES cell differentiation [25, 26]. Recently, SB431542 presents to increase neural induction ability in an embryoid body-based neural induction protocol from hES cells by suppressing the Lefty/Activin/TGFb pathways [14, 27]. Although Noggin or SB431542 treatment can prompt the efficiency of neural induction, treatment alone is not valid for neural induction by converting hES cells under defined or adherent conditions [14].

Multipotential stem cells from hES offer great promise for cell replacement therapies. Better differentiation protocols are necessary for reducing undefined factors in order to investigate the potential of these approaches in neural cell production. However, the qualified procedure is scarce and detailed features of hNSCs originated from hES cells are still unclear.

Here, we developed a method to obtain hNSCs from hES cells, by which we could harvest abundant hNSCs in a relatively short time. Most hES cells differentiated into NSCs according to this protocol. Then, we characterized the separating NSCs by detecting the expression of marker protein and identified their differentiation potential into astrocytes and neurons. Finally, we analyzed the mitotic activity and cell division cycle ratio of hNSCs and found that these hNSCs were healthy populations. Our study will provide detailed characteristics of hNSCs and improve the knowledge of how to obtain more hNSCs from hES cells. Our study may shed light on the therapeutic potential of these cell populations for the treatment of brain injury and disease.

## 2. Materials and Methods

### 2.1. hES Cells and Cell Culture Condition

hES cells (H9) were cultured for 30–32 passages on mouse embryonic fibroblasts (MEF) cells. MEF cells were obtained from 16-day pregnant mice and cultured in 10% foetal bovine serum (FBS) in high glucose DMEM (Gibco). Before using, MEF cells were treated with mitomycin C to block the MEF division. hES cells were planted on MEF cells at 1.8 × 10^3^/cm^2^ densities. And then, hES cells were cultured on MEF cells under the following conditions: DMEM/F12 (Gibco) containing 20% knockout serum replacement (Gibco), 1 × nonessential amino acids (Gibco), 1 × GlutaMAX (Gibco), 1/2 × penicillin/streptomycin (Gibco), 0.1 mM beta-mercaptoethanol (Sigma), and 6 ng/mL FGF-2 (HumanZyme) on 6-well plates. Medium was changed freshly every day. When cells needed to passage every 8 days, we used 1 U/mL dispase II (Roche) in DMEM/F12 (Gibco) for digesting the cells. Finally, we seeded the cells at 1 : 9 proportions into new 6-well plates.

### 2.2. Matrigel, Poly-L-ornithine, and Laminin-Coated Plates

The plates were coated with gelatin (Sigma) or Matrigel (BD) for neural stem cell induction. Other plates were precovered with poly-L-ornithine (Sigma) and laminin (Thermo Fisher) for human NSC culture and passage. The dishes are freshly coated with gelatin or Matrigel and must be kept overnight at 4°C for better package effect. The dishes were treated by 0.5 *μ*g/mL poly-L-ornithine (dissolved in water) at room temperature for 16 hours. Then, we applied 1x PBS to wash the dishes. Finally, 5 *μ*g/mL laminin was added to the dishes for at least 16 hours. The coated dishes can be centrally stored in the refrigerator for −20 degrees, and before using them, we needed to thaw and discard the supernatant liquid.

### 2.3. Neural Stem Cell Induction

hES cells were digested by applying StemPro Accutase (Thermo Fisher) for 20 min at 37°C. Then, cells were collected and washed with hES cell growth medium. Cell sedimentation was centrifuged and collected. The cells were put into the gelatin-coated plates for 1 hour at 37°C. Because hES cells were suspended and the MEF cells were adherent in existence of ROCK inhibitor (Tocris), we could collect the suspended cells and separated hES cells from MEF cells. The nonadherent hES cells were washed and plated at 5 × 10^4^ cells/cm^2^ density on Matrigel-precoated dishes in MEF-conditioned medium (DMEM/F12 containing 20% suspended medium collected from MEF cells which were not treated by mitomycin C, 20% KSR, 1 × nonessential amino acids, 1 × GlutaMAX, 1/2 × penicillin/streptomycin, 0.1 mM beta-mercaptoethanol, 10 ng/mL FGF-2, and ROCK inhibitor). After 1 day, the medium containing ROCK inhibitor was changed. Single adherent hES cells expanded in cell medium until they were almost confluent. Fresh medium was changed every 2 days. Noggin (500 ng/mL, R&D) and TGF-beta inhibitor (10 mM, Tocris) were added to confluent cells.

Fresh medium was changed every 2 days using KSR medium. On the 6th day, TGF-beta inhibitor was removed from differentiation medium and changed the cells into the medium with 25% N2 and 75% KSR with 500 ng/mL Noggin. Two days later, 50% N2 medium and 50% KSR medium with 500 ng/mL Noggin were given to cells. After 2 more days, 75% N2 medium and 25% KSR medium with 500 ng/mL Noggin were brought to cells. Nearly after 10 days of differentiation, NSCs were cultured with 100% N2 medium (DMEM/F12/N2/B27/GlutaMAX/FGF/EGF/heparin/penicillin/streptomycin) for 1 day and then transferred into a 100% N2/B27 medium (DMEM/F12/N2/B27/GlutaMAX/FGF/EGF/heparin/penicillin/streptomycin). hNSCs were passaged every 5 days at a 1 : 4 ratio onto the poly-L-ornithine and laminin-coated plates.

### 2.4. Neural Stem Cell Character and Differentiation

hNSCs were cultured in 100 mm dishes and passaged to poly-L-ornithine and laminin-coated 24-well plates at the 4th passage in N2/B27 medium supplemented with FGF/EGF/heparin factors. Three days later, hNSCs in 24-well plates were fixed with 4% paraformaldehyde (PFA) for 12 minutes.

To characterize the potential ability of NSCs, spontaneously differentiation was initiated. Spontaneously differentiation assay could induce NSCs into neurons, astrocytes, and oligodendrocytes. hNSCs were planted in the poly-L-ornithine and laminin-coated 24-well plates at a lower density in 100% N2/B27 medium with 3 factors (FGF/EGF/heparin) for 16 hours. On the second day, we changed fresh N2/B27 medium without FGF, EGF, and heparin factors. Then, we refreshed the medium without factors every 2 days. This process of spontaneous differentiation lasted for 21 days at least. Then, differentiated cells were fixed by using a method which was described above.

### 2.5. Antibodies, Immunostaining, and Microscopy

For BrdU immunostaining, the living cells needed to incubate with growth medium including 10 *μ*M BrdU for 24 hours in the dark CO_2_ incubator and then fixed cells by 4% PFA in PBS as follows. Fixed cells were permeabilized by 1% saponin in PBS for 8 minutes and then washed by 0.1% saponin in PBS. 2 mol/L HCl (2N) treated the cells for 18 minutes. Cells were washed by 0.1% saponin in PBS for three times and blocked with 5% bovine serum albumin (BSA) for 1 hour at room temperature. Cells were incubated with BrdU primary antibody at 4°C for 48 hours and second antibody at room temperature for 1.5 hours according to the general process.

For other antibody staining, cells fixed by 4% PFA were washed with PBS for 3 times and then were permeabilized with 2.5% Triton X-100. Cells were blocked with 5% BSA for 1 hour at room temperature. According to the general immunofluorescence technique, cells were washed with 0.1% Tween-20 in PBS and incubated with primary antibody at 4°C for 48 hours. Primary antibodies used for immunostaining were Oct4 (Biovision), Sox2 (R&D), Pax6 (Covance), Nanog (R&D), Nestin (R&D), Tuj1 (Covance), S100-beta (R&D), Ki67 (Thermo Fisher), and BrdU (Santa Cruz). The dilution buffer of primary antibody was 2.5% BSA in PBS. Cells were washed with 0.1% Tween-20 in PBS for 3 times and incubated with a second antibody at room temperature for 1.5 hours. At last, we used 4′,6-diamidino-2-phenylindole (DAPI, Sigma) to mark the nucleus of hNSCs. Additional attention, when we used DAPI, we must treat the cells for 10 minutes at room temperature after the second antibody incubation.

Cells were observed by using an inverted fluorescence microscope (Nikon). The images were acquired under a color CCD camera and digitized by PC-based frame grabber. Then, photos were analyzed by ImageJ, which was a powerful image analysis software. Then, the data collected from ImageJ were calculated by Excel. Calculation results were input into GraphPad Prism 6 and then organized as charts.

### 2.6. Flow Cytometry and Statistical Analysis

Cells were collected after digestion and centrifuged at 1000*g*/min for 3 minutes. Cell pellet was washed with 3 mL PBS and gently suspended in 50 *μ*L PBS. Cells were dropped to 1 mL precooled 70% ethanol in PBS. Cells were kept at −20 degrees overnight. On the second day, tubes containing cells were centrifuged at 500*g*/min. The supernatant was discarded, and the cells were resuspended gently with 400 *μ*L PBS containing 0.5 *μ*g/mL Hoechst 33258 (Sigma) and 10 *μ*g/mL RNAse A. The cell tubes were kept at room temperature in the dark. Lastly, we added Pyronin Y to the tubes at the final concentration of 0.5 *μ*g/mL. After 20 minutes, tubes were placed on ice in the dark and analyzed by flow cytometry (BD). The results from flow cytometry were calculated and analyzed by FlowJo.

All results were showed as mean standard deviation of the mean (SD). Data were calculated by Excel and *p* value was measured for statistical significance of two-tailed Student's *t*-test.

## 3. Results and Discussion

### 3.1. Results

#### 3.1.1. Induction of Neural Stem Cell from hES Cells

**Figure 1:**
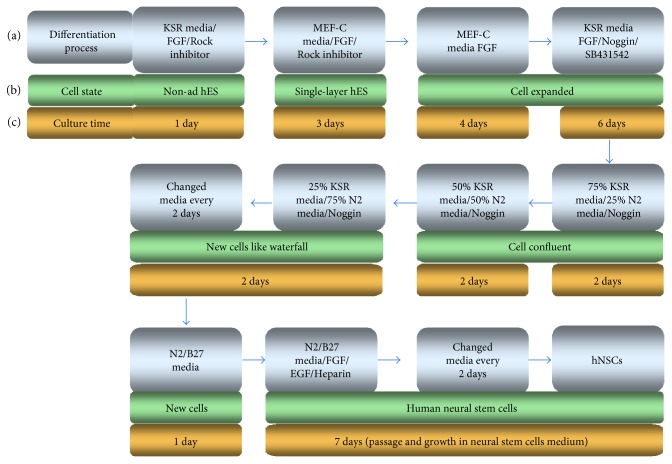
Flow chart of method used to obtain neural stem cell-like cells derived from human embryonic stem cells. (a) Differentiation process of human embryonic stem cells to neural stem cells. (b) State of cells in different differentiation stages. Human embryonic stem cells originally grew on feeder cells, and then cells were digested to single cell and removed the feeder cells. Single hES cells stuck to the dish bottom surface and grew to single-layer hES cells. Reduced medium and the factors were added to the single-layer hES cells. The cells were cultured until the end of differentiation. The process lasted about 4 weeks, and the cells were kept carefully in a fresh medim. KSR media: knockout serum replacement media; MEF-C media: mouse embryonic fibroblast cell conditional medium; non-ad hES: nonadherent human embryonic stem cells.

In order to develop an optimal scheme and detect the detailed features of hNSCs which differentiate from hES cells, we expanded the differentiation method to obtain hNSCs (Figure 1). Using this new protocol, hNSCs were isolated through a simple process and a relatively short time (Figure 1(a)). Concrete state of cells was showed during different differentiation stages (Figure 1(b)). During this differentiation process, cells needed to stay in a different medium for a specific culture time (Figure 1(c)). The overall process was hNSCs originally were cultured on MEF cells and were harvested to digest into single cells. After single ES cells grew to confluent layer, inducing factors were added to the confluent ES cells. Then, cells were cultured until the end of differentiation. The whole procedure lasted for 4 weeks, and during this period, it is necessary to continuously change medium details described as follows.

**Figure 2:**
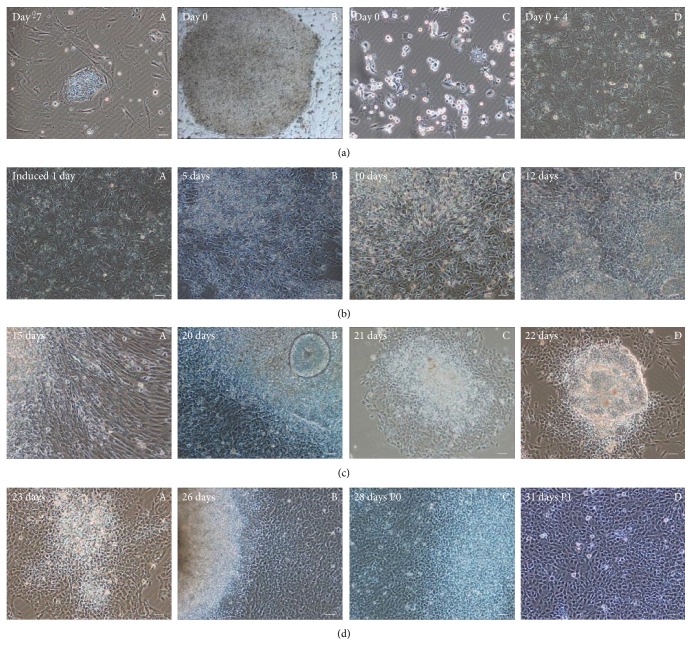
Neural stem cell-like cells were differentiated from human embryonic stem cells. (a) Human embryonic stem cells were cultured and digested into single cell, then the feeder cells (MEF cells) were removed, and single-layer cells were cultured in MEF conditional media until they were confluent. (A) hES cells grew on MEF feeder cells and will be digested after 7 days culture to start differentiation. (B) hES cells had grown on MEF feeder cells for 7 days and will be digested immediately. (C) hES cells were digested to gelatin-coated plates to remove MEF cells. (D) The nonadherent hES cells expanded in MEF-C medium about 4 days until confluent. (b) Noggin and SB431542 induced human ES cells into hNSCs. (A) The confluent single-layer hES cells were cultured in differentiation medium including KSR medium with TGF-beta inhibitor and Noggin for 1 day. (B) Differentiation for 5 days in differentiation medium. (C) Differentiation for 10 days and changing to KSR medium only with Noggin from the 6th day. (D) After 12 days, cells grew in 25% N2 media with 75% KSR medium for 2 days. (c) Cells crawled out from the assembled ES cells. After cell passage, the clone was visible. (A) Cells climbed from hESCs at 15 days. (B–D) State of cells around passage. (d) Differentiated cells from human ES grew homogeneously and fast. Scale bar = 50 *μ*m. (A, B) Different generations from passage 0 to passage 1.

With the purpose to demonstrate the process of inducing differentiation in detail, we documented various stages of cell differentiation. hES cells were cultured and digested into single cell as showed in the first two pictures (Figure 2(a)). In order to get enough cells, hES cells were cultured and amplified on MEF feeder cells (Figure 2(a), A). hES cells had grown on MEF feeder cells for 7 days and will be digested immediately (Figure 2(a), B). For removing MEF feeder cells, hES cells were digested with gelatin-coated plates (Figure 2(a), C). Nonadherent hES cells are planted into Matrigel-coated dishes and expanded in MEF-C medium about 4 days until confluent (Figure 2(a), D).

Cell morphology was presented during induction progress especially at days 1, 5, 10, and 12 with Noggin and SB431542 added to human ES cells (Figure 2(b)). Confluent single-layer ES cells were cultured in differentiation medium including KSR medium with TGF-beta inhibitor and Noggin for 1 day (Figure 2(b), A). State of Cells was showed after 5 days differentiation (Figure 2(b), B). Cells were differentiated for 10 days and changed to KSR medium only with Noggin from the 6th day (Figure 2(b), C). After 12 days, cells grew in 25% N2 media with 75% KSR medium for 2 days (Figure 2(b), D).

To track the cell differentiation state, we used imaging to record the birth of new cells during differentiation. Cells could be observed that there were many cells crawling out from the assembled hES cells (Figure 2(c)). Then, we passaged cells into growth medium which was prepared for hNSCs (Figure 2(c)). A large number of newborn cells climbed from hES cells in 15 days and 21 days (Figure 2(c), A-B). After cell passage, the hNSCs clone formed as showed (Figure 2(c), C-D). Differentiated cells from hES cells grew homogeneously and looked very healthy (Figure 2(d)). The morphology of cells may suggest that the new born cells had a healthy state and good proliferative ability (Figure 2(d), A-B). Different generations were shown from passage 0 to passage 1 (Figure 2(d), C-D).

### 3.2. Cells Differentiated from hES Cells Were Sox2 and Pax6 Positive

**Figure 3:**
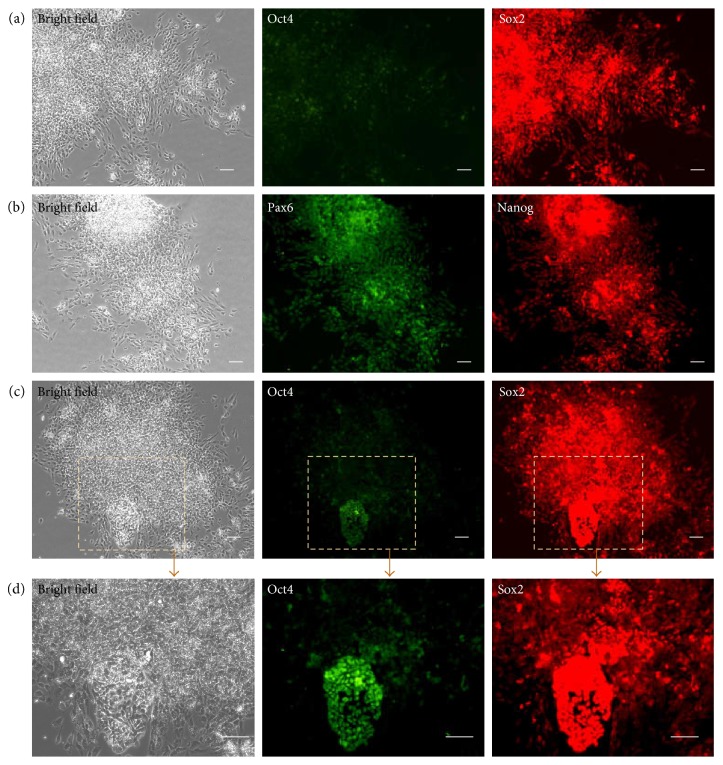
Human neural stem cell-like cells differentiated from hES cells were Sox2 and Pax6 positive. (a) Cells differentiated from hES cells were Oct4 negative and Sox2 positive. (b) Pax6 and Nanog were also expressed in human neural stem cell-like cells. (c, d) Most hES cells differentiated into hNSCs, but a small population hES cells did not differentiate into human neural stem cell-like cells and also showed embryonic cell character—Oct4. (d) It was an enlarged field of (c). Scale bar = 50 *μ*m.

To identify the differentiation tendency and potential of cells from embryonic stem cells, we used specific markers to test cell properties. We found that cells were Octamer-binding transcription factor 4 (Oct4) negative and Sox2 positive (Figure 3(a)). Oct4 was critically involved in the self-renewal of undifferentiated embryonic stem cells [28]. SRY- (sex-determining region Y-) box 2, also known as Sox2, is a transcription factor that is essential for maintaining embryonic and neural stem cells [29]. Nanog is a transcription factor critically involved with self-renewal [30]. Pax6 achieves its key roles in neurogenesis and proliferation [31]. Here, we found hNSCs expressed Pax6, the neurogenesis factors (Figure 3(b)). However, the expression of Nanog was decreased but still not fully withdrawn (Figure 3(b)). Persistence of transcription factor Nanog may be due to its critical role in the self-renewal of neural stem cells as well as in embryonic stem cells. In our study, although most hES cells could differentiate into hNSCs, there were a small population hES cells that refused to differentiate into hNSCs (Figure 3(c)). These little undifferentiating hES cells also showed embryonic cells character—Oct4 positive (Figure 3(d)) and died soon in the specific culture in hNSCs medium (data not shown).

### 3.3. Cells Expressed Marker Proteins of NSCs and Could Differentiate into Astrocyte and Neuron

**Figure 4:**
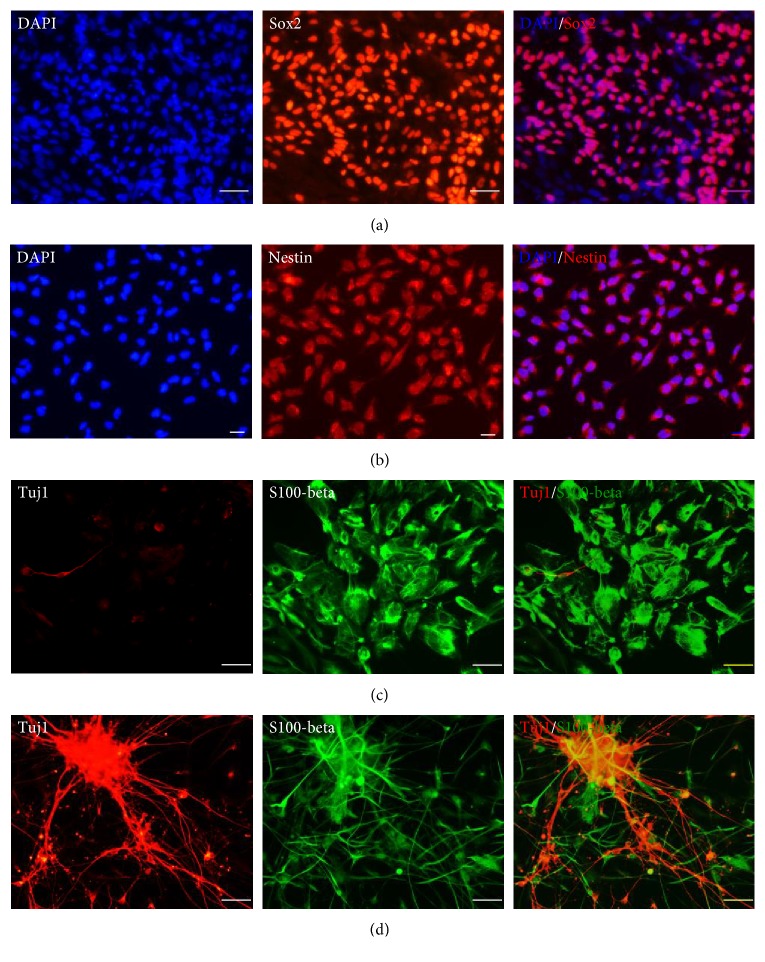
Cells expressed neural stem cell marker proteins and can differentiate into astrocyte and neuron. (a, b) Cells differentiated from hES cells expressed Sox2 and Nestin protein. (c, d) Cells express neural stem cell marker proteins and can differentiated into S100-beta-positive cells (astrocyte) and Tuj1-positive cells (neuron). (a) Scale bar = 50 *μ*m; (b–d) scale bar = 25 *μ*m.

To thoroughly investigate neural stem cells, we used the neural stem cell marker protein antibodies for immunostaining. We fixed the 4th passage cells differentiated from hES cells and discovered that these cells were Sox2 (Figure 4(a)) and Nestin (Figure 4(b)) positive. Cells expressed that both Sox2 and Nestin were considered as the characteristics of hNSCs. In order to detect the potential of cell differentiation, we used spontaneous differentiation to identify whether hNSCs could generate into glial cells and neurons. We found that cells could differentiate into S100-beta-positive cells (astrocyte) (Figure 4(c)) and neuron-specific class III beta-tubulin- (Tuj1-) positive cells (neuron) (Figure 4(d)).

### 3.4. Mitotic Activity Analysis of hNSCs

**Figure 5:**
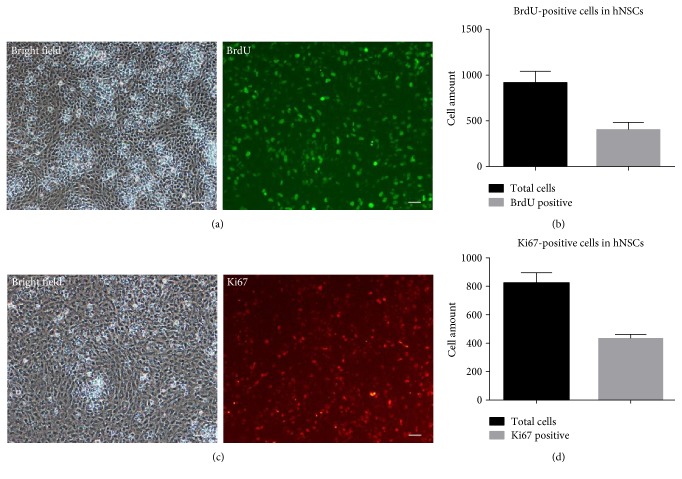
Mitotic activity analysis of human neural stem cells by using BrdU and Ki67 antibodies. (a) BrdU-positive cells were observed by immunofluorescence staining. (b) Nearly 40.2% (396/985) cells were BrdU-positive in hNSCs. (c) Ki67-positive cells were observed by immunofluorescence staining. (d) Nearly 52.2% (427/817) cells are Ki67-positive in hNSCs. Scale bar = 50 *μ*m.

To analyze the activity of cells, we utilized 5-bromo-2′-deoxyuridine (BrdU) antibodies to detect the division status of neural stem cells. BrdU is always used as a thymidine analogue in the identification of DNA synthesis. BrdU-positive cells were observed by immunofluorescence staining (Figure 5(a)), and almost half of the cells (40.2%) were BrdU positive in hNSCs (Figure 5(b)). Ki67, a marker protein of ribosomal RNA transcription, is a nuclear protein that is necessary for cellular proliferation. We also explored Ki67 to further investigate the cell proliferation. Data showed that Ki67-positive cells were observed by immunofluorescence staining (Figure 5(c)) and nearly half of the cells (52.2%) were Ki67 positive in hNSCs (Figure 5(d)).

### 3.5. Cell Division Phase Assay of hNSCs

**Figure 6:**
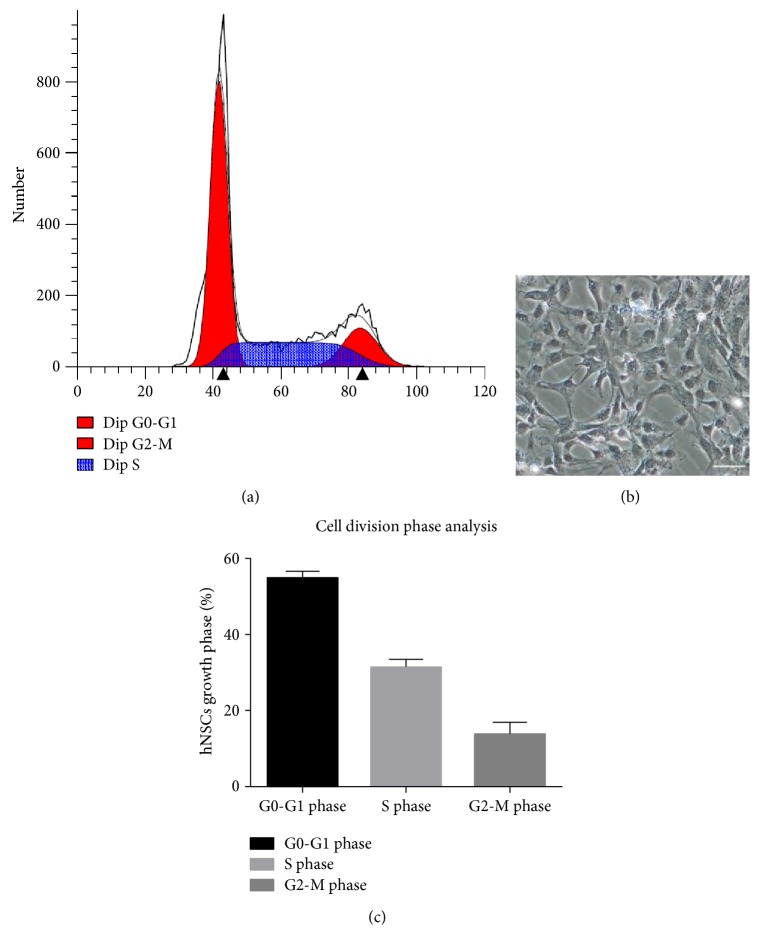
Flow cytometry analysis of human neural stem cells by using Pyronin Y and Hoechst 33258. (a) Flow cytometry analysis showed hNSCs and almost half of the cells were in division. (b) The morphology of hNSCs before flow cytometry. (c) Cell division phase analysis of hNSCs. 54% cells were distributed in G0-G1 phase, 31% in S phase, and 14% in G2-M phase. Scale bar = 50 *μ*m.

To obtain the character of cell division and cell phase, we extinguished hNSC flow cytometry analysis using Pyronin Y and Hoechst 33258 staining. Hoechst 33258 is a blue fluorescent dye using to stain DNA. Pyronin Y is used for RNA staining. G0/G1 phase DNA always kept at 2N but RNA started to replicate in G1 phase. DNA of S phase cells changed between 2N and 4N, and RNA continued to replicate. DNA content reached to 4N in G2/M phase. Almost half of hNSCs were in division (Figure 6(a)). Morphology of hNSCs was shown before flow cytometry analysis (Figure 6(b)). Data showed that 54% of cells were distributed in G0-G1 phase, 31% in S phase, and 14% in G2-M phase (Figure 6(c)).

## 4. Discussion

Our study provides detailed characteristics of hNSCs and improves the knowledge of how to obtain more high-quality hNSCs from hES cells. These results may help to prompt the therapeutic potential of these cell populations for cell therapy.

In this paper, we adopted a double-inhibiting method to obtain hNSCs from hES cells by combined applying of Noggin and SB431542. We harvested hNSCs through a simple procedure in a relatively short time. We identified the potential of cells from embryonic stem cells. Most hES cells could differentiate into NSCs, except a small population of Oct4-positive hES cells. These little nondifferentiation hES cells died quickly in the following culture (data not shown).

Then, we identified hNSCs by detecting the expression of marker protein and confirmed their differentiation potential into astrocytes and neurons. Furthermore, we analyzed the mitotic activity and cell division cycle ratio of hNSCs and found these hNSCs were active residents. To examine the activity of cells, we exploited Ki67 and BrdU antibodies to detect the division of neural stem cells.

Currently, studies of promoting hES cells to neural differentiation are mostly focused on differentiation of mature functional neurons or neural crest stem cells for cell therapy [15, 16]. Such differentiation procedure is developed for efficient production of dopamine progenitors from hES cells [16]. GABA neurons and cerebral cortex-specific neurons are derived from hES cells [17, 18]. At the same time, mature cortical neurons generated from hES cells by some small molecules [19–22]. Although the above studies can model cortical development fine, most of the cells which are produced from hES cells are a mixed population including mature neurons.

It is indistinct whether highly enriched hNSCs which had high self-renewal and proliferation capabilities have been generated from hES cells. Here, we developed differentiation protocols which eliminate the use of undefined factors. The defined differentiation factors will reduce the application obstacles of hNSCs in cell therapy. We investigated an efficient and fast differentiation approach to obtain hNSCs from hES cells successfully through a simple process in a shorter time compared to the usual method [25, 32].

Then, we identified the separating hNSCs by detecting the expression of marker protein and identified their differentiation potential into astrocytes and neurons. Finally, we analyzed the mitotic activity and cell division cycle ratio of hNSCs and found that these hNSCs were healthy populations. From hES cells to hNSCs, our differentiation method only needs about 3 weeks (from Figure 2(a),B) to Figure 2(c),C). Three weeks is a very short time because of previous reports. It frequently takes 5–7 weeks when inducing differentiation in the presence of MEF cells [25, 32].

Brain injuries such as traumatic injury, ischemic stroke, Parkinson's disease, or other neurodegenerative disorders are major causes of death and disability in the worldwide and brought serious social and economic burden [33–36]. Hence, brain injury therapies aimed to reduce neurological deficit are needed and cell therapies are crucial [37].

Stem cell transplantation plays a great potential to reduce the disability and promotes brain function after central nervous system (CNS) trauma and disease [38, 39]. Preclinical data points out that those cell-based therapies can enhance brain repair and significantly improve functional recovery after stroke or other brain injury [40]. They were proved safe and efficient both in stroke experimental animal and stroke patients [41, 42].

Cell-based therapies especially stem cell therapy also have been tested in some neurological disorders and get hopeful results proposing that it is maybe an efficient stroke therapy [42]. The reagents which target to protect neural stem cells, cerebral endothelial cells, astrocytes, oligodendrocytes, and neurons also can improve neurological function after stroke [37].

NSCs are immature precursors of the CNS and on self-renewal and multipotential differentiation abilities. Hormonal and local factors can directly regulate their proliferation and differentiation capability [43]. Alteration in neurogenesis is associated with many neurological disorders. Research results show that NSCs can be a potential therapy for brain injury [43].

Noggin is a BMP antagonist and plays an important role in neural tube development [44, 45]. Recombinant Noggin in mammalian also performs neural-inducing role [24]. Recombinant Noggin has been applied to several different neural induction protocols for hES cell differentiation [25, 26]. Lately, the drug SB431542 presents to support neural induction from hES cells [27]. SB431542 destroys the Lefty/Activin/TGFb pathways by inhibiting activities of ALK4, ALK5, and ALK7 receptors [14]. Although Noggin or SB431542 treatment prompts the efficiency of neural induction, treatment alone is not valid for neural induction by converting hES cells under defined or adherent conditions [14].

Multipotential stem cells from hES cells offer great promise for cell replacement therapies. Better differentiation protocols are necessary for reducing undefined factors in order to apply these approaches for the production of neural cells. However, detailed features of hNSCs which differentiate from hES cells are still unclear.

We also did the oligodendrocyte differentiation assay for 3 weeks, and cells presented the phenotype of oligodendrocytes. Oligodendrocyte differentiation medium consists of neurobasal medium supplementation with B-27, GlutaMAX-I, and T3. But finally, our immunostaining test did not obtain the ideal results and maybe, we need more tests. There was a difference between Ki67 and BrdU detecting division of neural stem cells. BrdU was generally used as a thymidine analogue in the identification of DNA synthesis. Proliferation marker protein Ki67 stops chromosomes from collapsing into a single chromatin mass and acts as a biological surfactant to separate mitotic chromosomes [46]. We should design more assays to discover the proliferation information. Additional work will be required to identify the mechanisms after double inhibition of hNSC differentiation and needed to further decrease the quantity close to zero of undifferentiated hES cells.

## 5. Conclusions

Cell-based therapy can increase functional recovery and help neurological brain injury. NSCs can be a potential therapy for brain injury. Multipotential stem cells from hES cells provide great promise for cell replacement therapies. Better differentiation protocols are necessary in order to produce more neural stem cells for therapies. However, the qualified procedure is scarce and detailed features of hNSCs originated from hES cells are still unclear. In this study, we developed a procedure to get hNSCs from hES cells, by which we could harvest abundant hNSCs in a pretty short time. We provided comprehensive features of hNSCs and delivered the knowledge about how to obtain more high-quality hNSCs from hES cells. These results may help to accelerate the therapy by using these stem cells to treat brain injury.
